# Calculation of Ejection Fraction Using Cardiac Computed Tomography: Clinical Evolution, Reliability, and Technological Challenges—A Narrative Review

**DOI:** 10.3390/medicina62061084

**Published:** 2026-06-02

**Authors:** Simone Steffani, Mariagrazia Piscione, Dario Gaudio, Giorgia Meghnagi, Gianluca Guelfand Crignola, Luigi Asmundo, Corrado Tagliati, Mario Laudazi, Marcello Chiocchi

**Affiliations:** 1 Diagnostic Imaging Department, University of Rome Tor Vergata, 00133 Rome, Italy; 2 Department of Cardiology, Santissima Annunziata Hospital, ASL2, 66100 Chieti, Italy; 3 Fondazione Policlinico Campus Bio-Medico, University of Rome, Alvaro del Portillo 200, 00128 Rome, Italy; 4 Department of Cardiology, Hospital F. Spaziani, Via Armando Fabi, 03100 Frosinone, Italy; 5 Department of Radiology, Ospedale Ca’ Granda, Piazza Ospedale Maggiore, 20162 Milano, Italy; 6 AST Ancona, Ospedale di Comunità Maria Montessori di Chiaravalle, Via Fratelli Rosselli 176, 60033 Chiaravalle, Italy

**Keywords:** cardiac computed tomography, ejection fraction, trans-thoracic echocardiography, cardiac magnetic resonance, pre-procedural planning, artificial intelligence

## Abstract

*Background*: The Ejection Fraction (EF) represents a fundamental pillar for the phenotypic classification and clinical management of cardiovascular diseases. Although trans-thoracic echocardiography (TTE) acts as the first-line examination and cardiac magnetic resonance (CMR) is the reference gold standard, cardiac computed tomography (CCT) has undergone a technological evolution. The advent of wide-detector scanners and artificial intelligence (AI) models has enabled CCT to transition from a purely morphological tool to a modality capable of comprehensive, three-dimensional morpho-functional assessments. *Methods*: This narrative review evaluates the literature across Scopus, MEDLINE, and Web of Science regarding the calculation of biventricular function and EF using CCT. It provides an updated summary of current clinical applications, technological advancements, and comparative diagnostic reliability against TTE and CMR. *Results*: The CCT “one-stop-shop” concept allows for the simultaneous acquisition of anatomical data and systolic function metrics (EDV, ESV, SV, EF), optimizing clinical workflows at no additional cost. Being intrinsically three-dimensional, CCT bypasses the geometric assumptions and apical foreshortening artifacts typical of 2D-TTE, demonstrating high volumetric concordance with CMR. Nevertheless, structural limitations persist, primarily regarding ionizing radiation exposure, contrast media toxicity, dependence on heart rhythm stability, and lower temporal resolution compared to CMR. *Conclusions*: EF determination via CCT has achieved technical maturity and clinical validation. While it does not intend to replace TTE or CMR, it offers synergistic data when integrated with primary anatomical indications. Furthermore, AI integration has been shown to potentially automate this workflow, transforming CCT into an opportunistic screening tool for subclinical cardiac dysfunction.

## 1. Introduction

### 1.1. Definition and Clinical Relevance of the Ejection Fraction

**Figure 1:**
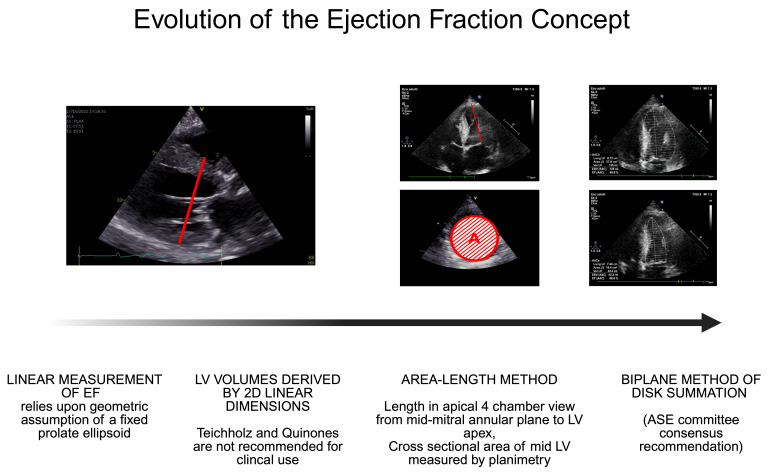
Evolution of the ejection fraction concept. Historical progression of TTE methods for calculating EF. The panel illustrates the transition from linear measurements relying on fixed geometric assumptions to the currently recommended biplane method of disk summation.

The Ejection Fraction (EF), primarily referring to the left ventricle (LV), represents the most widely used surrogate parameter for estimating global systolic function of the LV [1,2]. In modern cardiology, EF constitutes a useful parameter for the phenotypic classification of heart failure (HF), consequently guiding the pharmacological and interventional approach [3,4,5]. Beyond HF, accurate measurement of the pumping function is recommended in the assessment of valvular heart diseases, cardiotoxicity induced by oncological drugs, and right ventricular (RV) pathologies [1,6,7,8,9,10] (Figure 1). From a prognostic standpoint, even minor changes in EF values measured over time can reflect adverse remodelling capable of predicting major adverse cardiovascular events (MACE) [1,2,11,12,13]. Although EF is a composite parameter influenced by physiological determinants (such as preload, afterload, and ventricular geometry) rather than a direct measure of myocardial contractility, it continues to represent the most practical and widely adopted index for the assessment of global ventricular function in routine clinical practice [3,14].

### 1.2. Points of Strength and Weaknesses of the Use of the Ejection Fraction in Clinical Practice

**Figure 2:**
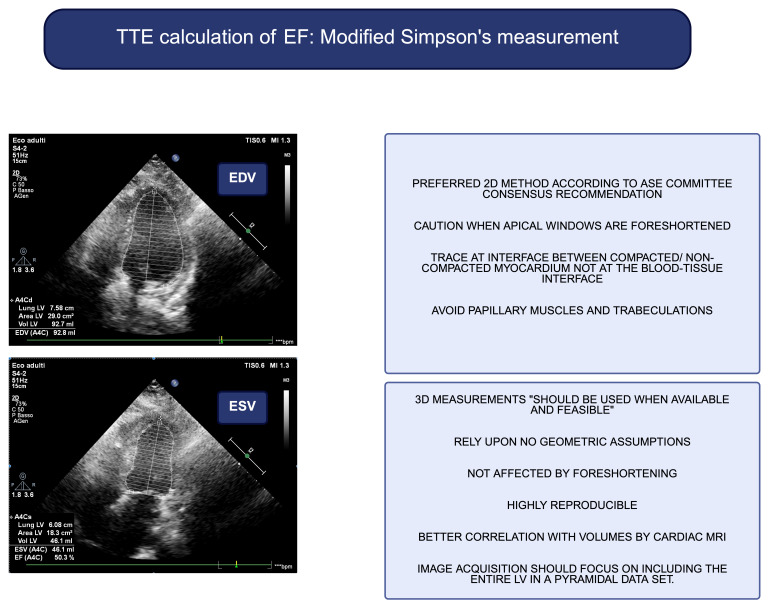
TTE Calculation of EF: Modified Simpson’s Measurement. Assessment of EDV and ESV using 2D-TTE according to the preferred biplane method of disks. The evaluation of these volumes is important, as LVEF represents a composite parameter that reflects both global ventricular structure and the overall haemodynamic state.

Traditionally, transthoracic echocardiography (TTE) has represented the first-line modality for EF assessment because of its wide availability, absence of radiation exposure, and real-time evaluation [4,15,16]. LVEF reflects the proportion of blood ejected during systole, but its value may vary significantly in response to loading conditions and ventricular remodeling [7,14]. Consequently, LVEF should be interpreted not only as an index of systolic performance but also as a marker of global ventricular structure and hemodynamic state [7] (Figure 2). Indeed, alterations in ventricular volumes or geometry can lead to apparently paradoxical LVEF values, since it is highly dependent on preload and afterload rather than being a direct measure of intrinsic myocardial contractility [7,14]. While advanced markers of myocardial function, such as indices of myocardial deformation (e.g., strain and strain rate), have been proposed to identify early myocardial impairment [1,15,17], LVEF continues to represent the most practical and widely adopted parameter for the assessment of global LV function in routine clinical practice [1,15,17,18]. However, TTE estimation of ventricular volumes and EF may be limited by operator dependency, suboptimal acoustic window quality, and geometric assumptions, suggesting the use of advanced three-dimensional imaging techniques in selected clinical scenarios [1,7,19].

### 1.3. CCT as a Versatile Alternative and Aim of the Review

**Figure 3:**
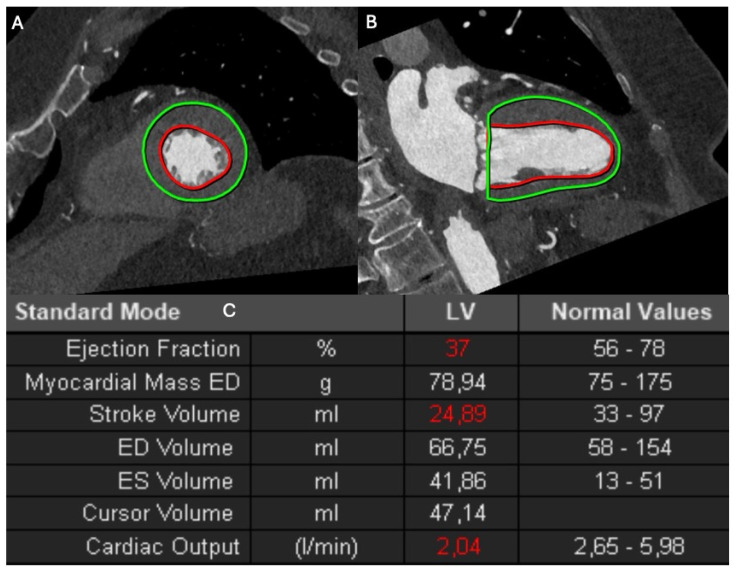
Left Ventricular Segmentation on Cardiac Computed Tomography. Representative multiplanar reformations illustrating the volumetric segmentation of the left ventricle across (**A**) a short-axis view and (**B**) a two-chamber long-axis view. The endocardial border is delineated by the red contour, while the epicardial boundary is outlined in green. (**C**) A resulting table demonstrating how automated border detection allows for the precise quantification of LV dimensions and functional parameters, including EDV, ESV, and SV. These datasets enable the calculation of myocardial mass and the derivation of the EF. The cardiac output is also calculated and expressed in liters per minute (L/min), providing a comprehensive assessment of cardiac performance.

In the last decade, advances in cardiac imaging technologies have established Cardiac Computed Tomography (CCT) as a diagnostic tool capable of providing high spatial resolution and three-dimensional volumetric datasets [2,20,21,22]. CCT allows for accurate quantification of ventricular volumes and EF without relying on geometric assumptions, offering both anatomical and functional information within a single examination [2,19,20,23]. Furthermore, CCT has been shown to potentially emerge as a viable alternative to CMR in evaluating functional metrics, especially when CMR is contraindicated or unavailable [2,9,22] (Figure 3) Therefore, the aim of this narrative review is not merely to restate that CCT can accurately measure EF, but to define precisely in which clinical scenarios it offers an added clinical value compared to standard modalities, and to critically highlight where its technological and clinical limitations still lie.

## 2. Evolution of CCT

Historically, the primary role of CCT in the thoracic and cardiac regions was purely morphological, confined to the evaluation of the Calcium Score (Agatston score) or the study of the coronary tree to exclude significant atherosclerotic stenoses [12,22,24]. However, overcoming temporal and spatial limitations thanks to new-generation multi-detector scanners has established a technological evolution [21,24]. Currently, CCT is no longer limited to the investigation of epicardial vessels but rather enables an integrated and three- dimensional morpho-functional assessment. Appropriately modulated retrospective or prospective acquisitions allow for sampling the entire cardiac cycle or targeted systo-diastolic windows [2,22,24,25]. The addition of advanced techniques, such as the evaluation of late iodine enhancement (LIE) or extracellular volume (ECV), provides tissue characterization that was previously the exclusive domain of CMR [2,22,26,27]. This evolutionary step has been shown to allow CCT to deliver anatomical, ischemic, and ventricular dynamics information in a single session, including myocardial deformation (strain) calculated through post-processing, especially through machine-learning technology [28,29,30].

## 3. Aim and Review Methodology

This narrative review aims to provide a comprehensive assessment of the published literature regarding the calculation of biventricular function, LVEF, right ventricular ejection fraction (RVEF), and atrial and ventricular volumes using CCT, with particular focus on current clinical applications, technological advancements, comparative diagnostic reliability, and emerging artificial intelligence–based approaches.

A structured bibliographic search was performed in Scopus (Elsevier, Amsterdam, the Netherlands) and MEDLINE (US National Library of Medicine, Bethesda, MD, USA) for studies published between January 2000 and January 2026. Combinations of the following descriptors were used according to a free-text search protocol: “cardiac computed tomography”, “ejection fraction”, “left ventricular volume”, “heart failure”, “pre-procedural planning”, “cardio-oncology”, “opportunistic screening”, “artificial intelligence”, “deep learning”, “wide-detector CT”, “echocardiography”, and “cardiac magnetic resonance”.

Only articles published in English were considered. Conference abstracts, editorials, commentaries, and isolated case reports were excluded. Particular attention was given to comparative imaging studies, technical validation studies, and clinically oriented investigations evaluating the role of CCT-derived functional assessment across different cardiovascular scenarios.

Titles and abstracts were independently screened by two authors (S.S. and M.P.) followed by full-text evaluation of potentially eligible studies. Discrepancies regarding study selection or relevance were resolved by consensus discussion among the authors. Given the narrative nature of the review, no formal quantitative synthesis or risk-of-bias assessment was performed. Overall, the final manuscript was based on a selective narrative synthesis of the studies considered most relevant to the objectives of the review, including methodological, technical, and clinically focused investigations.

## 4. Clinical Use of Ejection Fraction Measured by CCT

### 4.1. “One-Stop-Shop” Assessment

**Figure 4:**
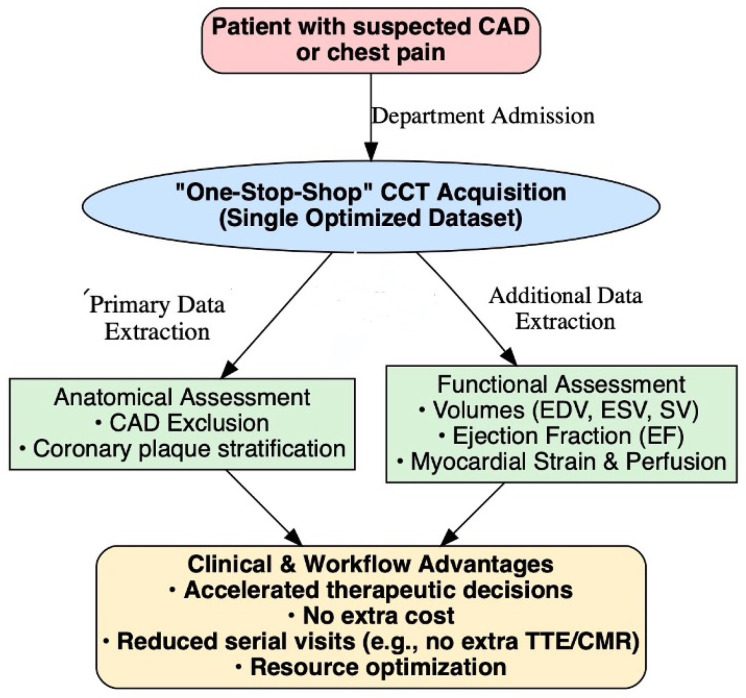
The “One-Stop-Shop” Concept in modern CCT.

The concept of “one-stop-shop” diagnostics (a single centre of diagnostic convergence) has found application in modern CCT [1] (Table 1). In patients presenting with suspicious chest pain or symptoms of ischemia, the primary objective is ruling out coronary artery disease (CAD) or stratifying the atheromatous plaque [12,25,31]. By exploiting datasets acquired in different phases of the cardiac cycle or retrospective reconstructions, it is, however, possible to simultaneously derive (although potentially requiring a slight increase in radiation dose compared to a single-phase CAD protocol, without further diagnostic delays) metrics of biventricular systolic function such as EDV, ESV, SV and, consequently, the global EF [24,25,31]. Beyond the purely clinical advantages, this integrated approach significantly impacts healthcare economics and workflow optimization [1,31]. Extracting pathophysiological information concurrently with primary anatomical evaluations provides added clinical data at no extra cost [2,21,25,31,32]. This dual capability reduces the hospital burden for the patient and optimizes resource allocation by eliminating the need to schedule multiple serial imaging tests (such as a TTE or CMR following the CCT) [19]. Consequently, this strategy reduces waiting times, minimizes the stress associated with multiple hospital appointments, and accelerates the therapeutic decision-making process [22,31,33]. A growing body of evidence supports the feasibility and clinical value of this integrated approach (Figure 4) [22]. In a recent study by Yunlong Hu and colleagues, a low-dose “one-stop” myocardial computed tomography perfusion (CTP) protocol was evaluated in patients with suspected CAD [22,34]. The authors demonstrated that this protocol allowed the simultaneous acquisition of coronary anatomical information, ventricular functional parameters, myocardial strain, and myocardial blood flow within a single CT examination [20,28,29,34]. Importantly, the one-stop protocol significantly reduced radiation exposure compared with conventional CCT protocols (4.13 ± 0.33 vs. 7.56 ± 1.43 mSv), corresponding to an approximate reduction of 44.5%, while maintaining comparable image quality metrics such as signal-to-noise and contrast-to-noise ratios [20,28,29,34]. Beyond the technical feasibility, the study also highlighted the functional and pathophysiological information obtainable from the same dataset [20,28,29,34]. LVEF and myocardial strain parameters showed a progressive deterioration with increasing severity of coronary stenosis, while myocardial blood flow values were lower in patients with significant CAD compared with those without obstructive disease [20,28,29,34]. These findings highlight how modern CCT protocols can integrate anatomical and functional data, enabling a comprehensive evaluation of CAD that extends beyond simple luminal assessment [20,28,29,34]. Such evidence further supports the concept that CCT may serve not only as an anatomical imaging modality but also as a platform for multiparametric cardiovascular assessment within a single examination [12,22].

### 4.2. Aetiological Diagnosis of New-Onset Heart Failure

**Figure 5:**
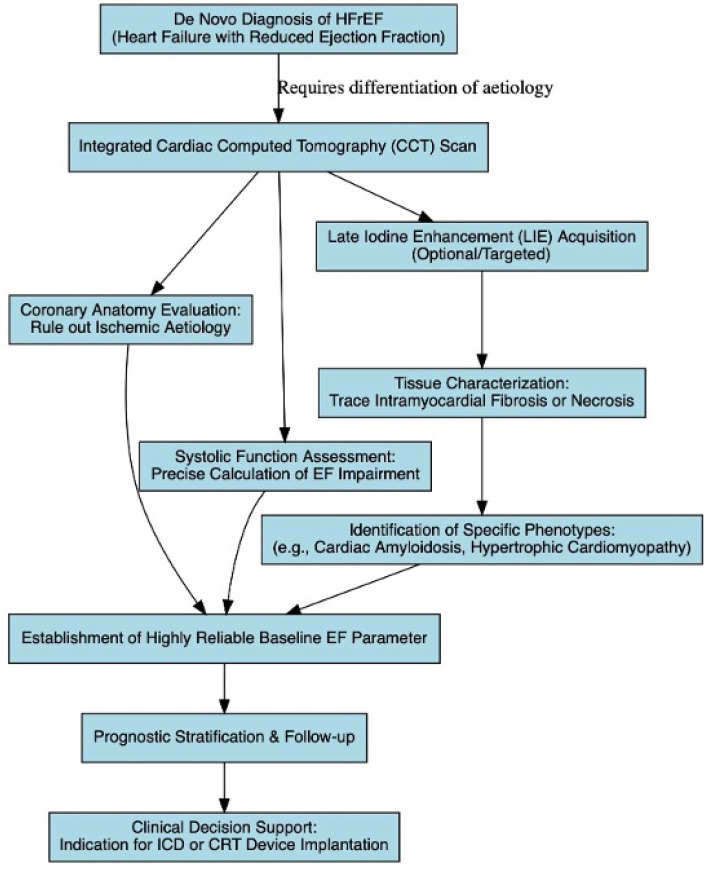
Integrated Cardiac Computed Tomography Pathway for the Etiological Diagnosis and Management of New-Onset HFrEF.

Faced with a de novo diagnosis of HFrEF, the approach suggested by major international guidelines is to differentiate an ischemic from a non-ischemic etiology (e.g., primary dilated cardiomyopathies, myocarditis, infiltrative cardiomyopathies) [5,22,35]. Historically, this process involved performing invasive coronary angiography combined with TTE or CMR [22,24]. Currently, the use of CCT in these scenarios may appear to be a valuable tool: in a single scan, the clinician can probe coronary integrity (excluding ischemic etiology of the disease) and precisely calculate the extent of EF impairment [20,22,24,31]. If the examination is implemented with LIE acquisition—albeit with intrinsically lower contrast sensitivity than gadolinium CMR—it is even possible to trace patterns of intramyocardial fibrosis or necrosis, guiding suspicion towards specific phenotypes (e.g., cardiac amyloidosis, hypertrophic cardiomyopathy, previous unrecognized ischemic insults) [20,22,26]. In this configuration, the EF provided by CCT acts as a highly reliable baseline parameter for follow-up and prognostic stratification, supporting the physician in the potential indication for the implantation of devices such as implantable cardiac device (ICD) or cardiac resynchronization therapy (CRT) device [36,37,38]. Figure 5 schematizes the integrated CCT pathway for the etiological diagnosis and management of new-onset HFrEF.

### 4.3. Diagnosis of Intraventricular Thrombosis in HFrEF

Beyond the evaluation of coronary anatomy and systolic function, CCT may also provide useful information in the detection of LV endocavitary thrombosis [20,24,39]. From a morphological standpoint, LV thrombi are typically located in the apical region and are generally contiguous with areas of akinetic or dyskinetic myocardium [40,41,42]. Unlike neoplastic masses, thrombi do not infiltrate the myocardial wall and usually appear as intracavitary structures adherent to the endocardial surface [40,41,42]. Their morphology may vary considerably depending on the stage of thrombus organization. Acute or recently formed thrombi tend to appear mobile, protruding, and relatively echo-lucent on TTE, often projecting into the ventricular cavity [43]. These mobile thrombi are associated with a higher risk of embolic complications [2,41,42,43]. In contrast, chronic or organized thrombi typically appear laminated, sessile, and more echogenic, with a smoother surface and a morphology that may resemble hepatic tissue on ultrasound imaging [43]. TTE remains the first-line imaging modality for the detection of intraventricular thrombi because of its wide availability, non-invasive nature, and ability to simultaneously assess LVEF and regional wall motion abnormalities [10,42,43,44]. Several TTE parameters have been shown to correlate with thrombus formation, including severely reduced LVEF (typically <40%), high wall motion score index, and elevated diastolic filling pressures reflected by increased E/E′ ratio [45]. However, despite these advantages, the sensitivity of standard TTE for detecting LV thrombus may be limited, particularly in patients with suboptimal acoustic windows or when differentiating thrombus from prominent trabeculations, apical muscle bands, or spontaneous echo contrast [7,45,46]. In such cases, the use of intravenous contrast agents significantly improves endocardial border delineation and enhances diagnostic accuracy by allowing the thrombus to appear as a filling defect surrounded by contrast-enhanced blood pool [7,41,42]. Advanced 3D TTE techniques such as three-dimensional imaging may improve visualization by providing volumetric datasets that allow the intracavitary mass to be analysed from multiple perspectives, although this technology is not universally available [7,19,47]. In this context, CCT has emerged as a potentially valuable complementary imaging modality for the detection of LV thrombus [7,42,45]. The high spatial resolution of CCT and the ability to obtain multiphase datasets enable detailed visualization of intracavitary structures and accurate differentiation between thrombus, trabeculations, and slow-flow artefacts [20,21,42]. On contrast-enhanced CT, LV thrombus typically appears as a low-attenuation filling defect within the contrast-opacified ventricular cavity, clearly separated from the surrounding blood pool. This contrast differentiation allows improved identification of mural thrombi that may otherwise be difficult to detect with TTE [20,22,24].

### 4.4. CCT to Assess of Right Ventricular Ejection Fraction (RVEF)

**Figure 6:**
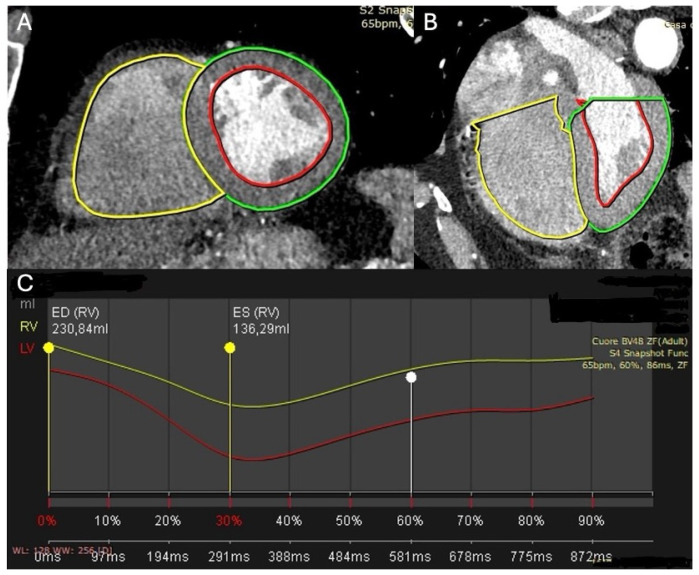
Biventricular Segmentation on Cardiac Computed Tomography. (**A**) Representative multiplanar reformations illustrating the volumetric segmentation across short-axis and (**B**) four-chamber view. The LV endocardial border is delineated by the red contour, while the epicardial boundary is outlined in green. The RV endocardium is segmented with a yellow line. Automated border detection allows for the precise quantification of biventricular dimensions and functional parameters. (**C**) The accompanying table displays the EDV and ESV for both the left and right ventricles. These datasets enable the calculation of myocardial mass and the derivation of the EF and SV, providing a comprehensive assessment of cardiac performance.

The RV is a highly adaptive chamber whose structure and function are strongly influenced by loading conditions and ventricular–vascular interactions [9,48,49]. In the presence of chronic hemodynamic stress, the RV undergoes a progressive remodelling process characterized by chamber dilatation and gradual impairment of myocardial contractility [48,49,50,51]. Over time, this adaptive response may evolve into a state of advanced RV dysfunction, in which the ability of the ventricle to generate effective forward SV becomes progressively compromised [44,48,52,53]. The evaluation of RV systolic function remains particularly challenging because of the complex crescent-shaped geometry of the ventricle and the marked load dependence of conventional functional indices [9,53,54]. TTE represents the first-line imaging modality for assessing RV size and function; however, traditional parameters such as tricuspid annular plane systolic excursion and fractional area change reflect only specific components of RV contraction and may not fully capture global ventricular performance [52,55]. These indices are highly sensitive to loading conditions and may therefore provide an incomplete representation of intrinsic myocardial contractility [8,9,56]. Advanced imaging techniques have improved the characterization of RV mechanics. Two-dimensional speckle-tracking TTE enables the assessment of RV free-wall longitudinal strain, which has emerged as a sensitive marker of myocardial dysfunction and has been shown to detect early impairment beyond conventional TTE indices [9,15,54]. More recently, CCT has demonstrated the ability to provide reliable volumetric quantification of RV function [57]. Functional CCT datasets permit the measurement of RV volumes and RVEF with good reproducibility, offering an additional tool for the assessment of RV performance [22,46,49,57]. Notably, CCT-derived RVEF has been shown to correlate with clinical outcomes in several clinical contexts, supporting the potential role of CCT-based functional assessment as a complementary parameter in the evaluation of RV remodelling and prognostic stratification [13,51,57] (Figure 6).

### 4.5. CCT for Advanced Pre-Procedural Planning

An area where the calculation of EF by cardiac CCT has assumed relevant application is the pre-procedural planning of interventional procedures on valves and congenital heart diseases [21,22]. In Transcatheter Aortic Valve Implantation (TAVI) procedures, CCT is already widely used to study vascular accesses, the aortic root, the height of the coronary ostia, the grading of valvular calcium and the sizing of the future prosthesis [19,21,23,25,58]. However, by extracting information on EF and ESV and EDV from the same dataset, the Heart Team is able to quantify the extent of cardiac damage induced by severe aortic stenosis, a parameter known to be strongly predictive of short- and long-term outcomes post-valve replacement [17,25,26]. Similarly, in scenarios of mitral regurgitation evaluated for transcatheter replacement devices, CCT provides not only annular dimensions and leaflet relationships to avoid systolic anterior movement but also yields an objective, highly three-dimensional estimate of left ventricular volumes [26,58,59].

### 4.6. The Emerging Role in Opportunistic Screening and Cardio-Oncology

Although EF is a strictly cardiological parameter, its evaluation assumes clinical importance in systemic contexts, primarily cardio-oncology. The advent of highly effective antineoplastic therapies, such as anthracyclines (e.g., doxorubicin) and HER2 inhibitors (e.g., trastuzumab), has concurrently increased the incidence of drug—induced cardiotoxicity, defined as cancer therapy-related cardiac dysfunction (CTRCD) [6,47,60]. Traditionally, serial surveillance of EF in these patients has been entrusted to radionuclide ventriculography (MUGA scan) or TTE [10,31,47,60]. More recently, CMR has been used as the reference method due to its high reproducibility, which is fundamental when significant, guideline-defined declines in EF (e.g., >10 percentage points from baseline to values <50%) dictate the suspension of life-saving drugs [2,10,11]. However, the vast majority of cancer patients regularly undergo CCT of the chest, abdomen, and pelvis for tumor staging and the evaluation of therapeutic response [31,61]. Here, the concept of “opportunistic screening” comes into play: extracting cardiac functional data from CT scans performed for primarily non-cardiac indications [6,62,63]. While pure opportunistic screening relies on the application of emerging AI models on standard non-gated scans, some institutions are also proactively modifying their staging protocols [32,63,64,65]. Implementing specific ECG-gated acquisitions in optimized chest CT protocols allows for the calculation of biventricular EF without subjecting the oncology patient to further investigations (such as TTE or CMR), reducing waiting times [31,66]. Furthermore, adding a late acquisition to these modified protocols enables the calculation of ECV in the septal myocardial layer during the delayed phases rather than the standard venous phases of the staging study; an early increase in ECV on CCT has proven to be a biomarker capable of anticipating the frankly systolic decline of EF in breast cancer patients treated with anthracyclines, acting as a warning bell for interstitial remodeling and reactive fibrosis [6,67,68].

**Table 1:** Summary of Clinical Scenarios and the Added Value of CCT-Derived Functional Assessment.

**Clinical Scenario**	**Primary Indication for CCT**	**Added Value of Functional Assessment (Volumes and EF)**
Suspected CAD (“One-stop-shop”)	Ruling out coronary artery disease or stratifying the atheromatous plaque [22,65].	Simultaneous derivation of biventricular systolic function (EDV, ESV, SV, EF) optimizing workflow and accelerating therapeutic decisions at no extra cost [24,33,51,69].
New-onset HFrEF	Differentiating ischemic from non-ischemic aetiology and tracing patterns of intramyocardial fibrosis/necrosis via LIE [22,27,70,71].	Establishes a highly reliable baseline EF for prognostic stratification and to guide ICD or CRT device implantation [2,7,36,37].
Intraventricular thrombosis (HFrEF)	Complementary imaging when standard TTE sensitivity is limited by suboptimal acoustic windows [23,42].	High spatial resolution enables detailed visualization and accurate differentiation between thrombus, trabeculations, and slow-flow artifacts [20,24]
Right ventricular assessment	Assessing RV size and function when traditional TTE parameters are limited by load dependence and complex geometry [9,51]	Provides reliable volumetric quantification of RV volumes and RVEF, correlating with clinical outcomes and RV remodelling [9,51]
Advanced pre-procedural planning	Evaluating vascular accesses, aortic root, valvular calcium, and prosthesis sizing for TAVI or mitral interventions [21,23,26].	Extracts EF, EDV, and ESV to quantify cardiac damage, which is strongly predictive of post-operative outcomes [26,59].
Opportunistic screening and Cardio-Oncology	Routine chest, abdomen, and pelvis CT for tumour staging and therapeutic response evaluation [31,32]	Calculation of biventricular EF from non-cardiac scans (via AI or modified ECG-gated protocols) avoiding extra TTE/CMR to monitor cardiotoxicity [6,22,31].

## 5. Technological Challenges: Acquisition, Wide-Detector Scanners, and Artificial Intelligence

### 5.1. Hardware Evolution: The Impact of 16 cm Scanners (Wide-Detector CT)

The reliability of volumetric and functional calculations via CCT inherently depends on the spatial and temporal resolution of the equipment [22,24,46]. Historically, 64-slice scanners required multiple rotations of the X-ray tube and the acquisition of data over several cardiac cycles to cover the entire longitudinal extension of the heart (approximately 12–14 cm) [1,24,38,72]. This modality, especially in retrospective protocols necessary to reconstruct the entire R-R cycle in 5–10% intervals, exposed the patient to considerable radiation doses and stair-step artifacts in cases of heart rate variability or arrhythmias, irreparably invalidating the calculation of EDV and ESV [4,8,15,24,38,61]. The technological advancement was marked by the introduction of wide-detector scanners, equipped with a *Z*-axis coverage of up to 16 cm (e.g., 256- or 320-slice scanners) [24,28,30,38]. These devices allow whole-heart volumetric imaging in a single gantry rotation and within a single heartbeat [24,30,31,38,49]. The absence of spatial misregistration guarantees volumetric measurements of the LV chamber of high precision [38,64,72]. Even with prospective acquisitions limited only to the systolic and diastolic phases, which enable effective dose reductions below 1–2 mSv, the calculation algorithm returns highly accurate SV and EF values [13,31,61]. However, it must be noted that while dual-phase scans are sufficient for volumetric calculations, complex deformation (strain) analyses require a full multiphase acquisition; studies demonstrate that modern protocols covering the entire cardiac cycle (Low-Dose 4DCT) can excellently preserve endocardial kinetics for this specific purpose [14,19,25,29,33,73] (Table 2).

### 5.2. The Breakthrough of Artificial Intelligence and Deep Learning

Until the recent past, the calculation of EF on CCT was limited by time constraints: the operator had to manually or semi-automatically trace the endocardial contours (epicardial for myocardial mass) of the LV, slice by slice, from base to apex in end-systole and end-diastole [18,46,66]. Today, convolutional neural networks (CNNs) and U-Net architectures (e.g., 3D or 8-layer residual U-Net) have highly automated this workflow [28,29,66]. These AI models, trained on massive cohorts of validated examinations, can automatically segment the ventricular blood pool, excluding papillary muscles and trabeculae carneae from the cavitary volume or including them according to adopted conventions, with a degree of precision that may appear comparable or superior to a level 3 expert [31,46,66,68]. While standard clinical practice relies on contrast enhancement, emerging AI models are demonstrating the ability to estimate EF and even atrial volumes directly from non-contrast chest CCT scans (such as those performed for the Calcium Score or lung cancer screening) [13,63,65]. Specific algorithms (e.g., AI-CAC) exploit the slight radiological density gradient between the myocardium and the blood to delineate the cardiac chambers, generating predictive models capable of anticipating future hospitalizations for HF better than consolidated serological biomarkers such as NT-proBNP [15,64,75]. This level of automation transforms raw data into an immediate morpho-functional report, minimizing inter- and intra-operator variability under optimal heart rate conditions, although manual editing may still be required in cases of severe arrhythmias [12,18,51]. However, despite these promising advancements, it is important to maintain a balanced and critical perspective [65]. The notion that AI automation is fully ready for widespread, unsupervised implementation is premature [65]. Many current models depend heavily on selected datasets and optimal image quality, raising significant concerns regarding their generalization and cross-platform reproducibility in diverse, real-world clinical environments [21,65]. High technical performance in a controlled setting does not seamlessly translate into actual clinical utility [65]. Manual correction by an expert reader remains frequently necessary, particularly in the presence of severe arrhythmias, motion artifacts, or complex anatomies [12,21,68]. Therefore, AI should currently be viewed as a highly capable supportive tool rather than an autonomous solution [65,66,76]. Before these algorithms can become the standard of care, the scientific community must overcome substantial barriers, including the need for rigorous external validation across heterogeneous populations, seamless integration into existing hospital PACS workflows, and the stringent control of algorithmic biases [65].

### 5.3. Analysis of Diastolic Function and Atrial Volumes

While the EF serves as a relevant marker of systolic performance, the comprehensive phenotypic classification of HF (including HFpEF) mandates the evaluation of diastolic function [5,16,77,78]. The volumetric datasets acquired during CCT are not limited to ventricular chambers; they concurrently enable the precise measurement of maximum and minimum left atrial (LA) volumes, which are robust surrogate markers of chronicity in left ventricular diastolic dysfunction and elevated filling pressures [16,22,63]. Traditionally, LA volume quantification has been entrusted to TTE or CMR [16,58]. However, recent advancements have demonstrated that CCT, coupled with deep learning algorithms, can fully automate the extraction of left atrium and LA appendage volumes with high accuracy, seamlessly integrating these predictive models into standard clinical workflows [11,30]. By integrating atrial volumetry and morpho-functional parameters, CCT provides a holistic hemodynamic profile that bridges the gap between purely systolic metrics and the complex continuum of diastolic impairment, thereby enhancing the predictive value for adverse cardiovascular events [11,13,22].

## 6. Reliability: Comparison with Clinical Gold Standards (Echocardiography and CMR)

The diagnostic value of CCT-derived EF assumes real clinical weight only if analyzed in a head-to-head comparative perspective with the primary modalities of cardiovascular imaging: TTE (first-line standard) and CMR (reference standard).

### 6.1. Comparison with Transthoracic Echocardiography

While 2D-TTE represents the most accessible and widely adopted first-line modality, the calculation of EF is based on the biplane method of disks (modified Simpson’s rule) [1,14,19]. However, 2D-TTE remains limited by operator dependency, the assumption of standard ventricular geometry, and the frequent impossibility of obtaining optimal acoustic windows in specific clinical settings, such as obesity, severe emphysema, or mechanical ventilation [1,14,19,46]. In addition, artifacts such as apical foreshortening may lead to systematic underestimation of true ventricular volumes [14,19,46,79].

Being intrinsically three-dimensional, CCT overcomes many of the geometric assumptions inherent to 2D-TTE and generally provides higher EDV and ESV values, likely reflecting a more complete visualization of ventricular anatomy [23]. CCT has been shown to offer improved delineation of the apical and lateral endocardial borders, which can be difficult to visualize with echocardiography, even in the era of three-dimensional TTE [19,23,79,80]. Nevertheless, these differences should not be interpreted as evidence of universal superiority or interchangeability between imaging modalities, since volumetric agreement may vary according to image quality, acquisition protocols, rhythm stability, and patient-related factors [2,59,79,81]. Furthermore, fully automated deep learning–based CCT analysis demonstrated excellent reproducibility for cardiac functional assessment, with nnU-Net achieving Dice Similarity Scores of 0.91 and near-perfect agreement for LVEF estimation (ICC = 1.00) while also accurately quantifying LV stroke volume (ICC = 0.95) and left atrial function parameters [11].

Although discrepancies between CCT- and TTE-derived volumetric measurements have been associated with post-operative systolic dysfunction in selected valvular populations, these observations derive primarily from methodological or observational studies and require further validation before broad clinical generalization [59].

### 6.2. Comparison with Cardiac Magnetic Resonance

CMR with Steady-State Free Precession (SSFP) cine sequences represents a highly precise method, thanks to the high intrinsic contrast between the hyperintense blood pool and the medium-signal intensity myocardium, without the use of iodinated contrast media or ionizing radiation [8,19,22,49,60]. Compared to CMR, cardiac CCT has demonstrated high concordance, with Pearson correlation coefficients frequently exceeding 0.90 for EDV, ESV, and LVEF [2,13,33,51]. Supporting these observations, a large systematic review including 65 studies and 4032 imaging examinations demonstrated minimal volumetric bias between MDCT and CMR for LVEDV (−1.20 mL, *p* = 0.43) and LVESV (−0.13 mL, *p* = 0.91), indicating excellent agreement for ventricular volume quantification [79]. However, it is important to emphasize that this high correlation is primarily observed under strictly optimal conditions: stable sinus rhythm, appropriately tailored ECG-gated protocols, optimal contrast enhancement, and accurate image reconstructions [3,46]. In the absence of these prerequisites—such as in patients with severe arrhythmias or sub-optimal breath-holding—the reliability of CCT drops significantly [3,21,38]. Firstly, analyzing temporal resolution, modern CMR typically acquires between 30 and 50 phases per cardiac cycle [2,3,19,22,36]. CCT, conversely, generally divides the retrospective cycle into 10–20 phases (or targets predefined fixed intervals in dual-phase prospective protocols), presenting an effective temporal resolution of 66–140 ms, limited by the gantry rotation time [2,17,18,22,24,25,46,51,58]. It follows that CCT may “miss” the true end-systole and end-diastole frames (peak of maximum contraction and filling), leading to a slight overestimation of the ESV and potential underestimation of the EDV [13,19,20,31]. This technical discrepancy generates an inevitable repercussion on the EF: since EF is calculated as the ratio of stroke volume to EDV (EF = EDV-ESV/EDV × 100), an overestimation of the ESV coupled with a potential underestimation of the EDV by CCT mathematically translates into a slight systematic underestimation of the EF compared to CMR (on average by 2–4%) [13,18,66]. While the Bland–Altman limits of agreement in research settings appear sufficiently narrow for the macro-classification of pumping function, caution must be exercised before assuming true clinical interchangeability [47,79,82]. A strong mathematical correlation in methodological studies does not automatically equate to practical equivalence in day-to-day clinical decision-making, particularly when precise LVEF threshold values dictate critical interventions (e.g., ICD placement or withholding cardiotoxic chemotherapy). While CCT is a highly reliable alternative, its functional assessment should be contextualized within its technical boundaries [19,23,46] (Table 3).

## 7. Limitations and Issues of the Method

Despite technological progress, the routine implementation of CCT exclusively for EF calculation still encounters important clinical and technical limitations. Data suggest that the clinical utility of functional CCT depends not only on technological performance, but also on the clinical context and careful patient selection based on an appropriate risk-benefit balance [21]. In contemporary clinical practice, CCT is rarely performed solely for ejection fraction quantification [22]. Rather, functional assessment is generally obtained as an adjunctive component of examinations primarily indicated for anatomical evaluation, such as CAD exclusion, structural heart disease assessment, or pre-procedural planning, thereby allowing EF and volumetric parameters to be extracted as additional information without requiring further imaging tests [21,22]. Consequently, these limitations still prevent the use of CCT as a first-line stand-alone screening modality for routine functional assessment in the general population [27,47,63].

### 7.1. Exposure to Ionizing Radiation and Contrast Media Toxicity

The principal limitation of CCT remains exposure to ionizing radiation [21,22]. In earlier generations of scanners, retrospective acquisition throughout the entire cardiac cycle resulted in relatively high effective radiation doses (often exceeding 10–15 mSv), which were considered difficult to justify for the sole purpose of EF calculation [24,31,46]. The introduction of ECG-dependent dose modulation and prospective acquisition protocols has substantially reduced radiation exposure (frequently <2 mSv); nevertheless, stochastic biological risk persists, making CCT generally unsuitable as a first-line modality for serial follow-up examinations, particularly in young patients and women of childbearing age [21,22,24,31,61].

An additional limitation is the requirement for iodinated contrast administration [21,22]. In patients with advanced HF or cardio-renal syndromes, the risk of contrast-induced acute kidney injury (CI-AKI) may be increased [21,22]. Although deep learning approaches are currently exploring automated segmentation from non-contrast datasets, accurate delineation of the trabeculated endocardial border for routine clinical volumetric quantification still generally requires adequate blood-pool opacification [13,21].

Radiation exposure associated with CCT should also be interpreted in the context of other commonly used cardiovascular imaging modalities [31]. Historically, retrospective ECG-gated CCT protocols frequently exceeded 10–15 mSv, values comparable to or higher than those associated with invasive coronary angiography or nuclear imaging techniques such as SPECT myocardial perfusion imaging [19,31]. However, contemporary low-dose CCT protocols based on prospective ECG-triggering, ECG-dependent tube current modulation, iterative reconstruction algorithms, high-pitch acquisitions, and wide-detector scanners may reduce radiation exposure to approximately 1–3 mSv in selected patients, approaching or even falling below the dose range reported for MUGA studies and some diagnostic invasive angiographic procedures [31,61].

Nevertheless, radiation burden remains highly dependent on acquisition protocol, patient characteristics, heart rhythm stability, and scanner technology, suggesting the importance of individualized risk-benefit assessment before selecting CCT for functional evaluation [21,24].

### 7.2. Artifact Management and Heart Rhythm

The accuracy of ESV and EDV measurements is highly dependent on rhythm stability and adequate breath-holding during image acquisition [3,21,38]. Severe arrhythmias, atrial fibrillation with rapid ventricular response, and frequent premature beats may impair ECG synchronization and reduce the reliability of volumetric reconstruction [21,46]. Although wide-detector (16 cm) scanners have substantially reduced stair-step artifacts through whole-heart single-beat acquisition, marked beat-to-beat R-R variability may still generate non-diagnostic frames, particularly during systole, requiring motion-correction algorithms and manual editing that may prolong post-processing and reporting time [12,28,33,38].

### 7.3. Temporal Resolution

As previously discussed in the comparison with CMR, the temporal resolution of CCT remains intrinsically constrained by gantry rotation speed (currently approximately 210–280 ms in high-end systems, corresponding to an effective temporal resolution of approximately 66–140 ms depending on single- or dual-source technology) [19,24,33,46]. This remains inferior to both cine-CMR (typically 20–40 ms) and TTE, which can achieve extremely high frame rates corresponding to temporal resolutions of approximately 10–11 ms [19,49,56,81].

Consequently, CCT may not always capture the exact moment of maximal contraction or maximal filling, potentially leading to slight smoothing of volumetric peaks and small systematic deviations in ESV, EDV, and EF estimation [19,53]. Although these differences are often clinically modest, they may become more relevant in borderline scenarios where small EF variations can influence therapeutic decision-making [10,68].

## 8. Current Guideline-Based Clinical Positioning of CCT-Derived Functional Assessment

From a practical guideline-oriented perspective, it should be emphasized that no current major international guideline specifically recommends CCT for stand-alone EF quantification or routine serial functional assessment. Rather, contemporary recommendations primarily position CCT within diagnostic pathways focused on ischemia detection, anatomical characterization of CAD, and cardiovascular risk stratification. For this reason, the present discussion mainly refers to the most recent ESC 2024 and AHA/ACC 2023 guidelines on chronic coronary syndromes/chronic coronary disease, which better reflect the rapid technological evolution and expanding evidence base of CCT applications [83,84]. In contrast, earlier documents, such as the 2021 ESC Heart Failure Guidelines, assigned a more limited role to CCT in ischemic evaluation, partly because many of the contemporary validation studies and technological advancements currently available had not yet been published at the time of guideline development [85].

Within this evolving framework, the 2024 ESC Guidelines assign a Class I recommendation (LOE B) to CCTA as an initial diagnostic test in symptomatic patients with low-to-intermediate likelihood of obstructive CAD, while the 2023 AHA/ACC Chronic Coronary Disease Guidelines support a complementary use of CCTA, particularly for risk stratification and selected anatomical evaluations [83,84]. Neither guideline currently recommends CCT-derived EF assessment as a dedicated primary indication [83,84]. Nevertheless, when CCT is already clinically indicated, the simultaneous extraction of functional information—including ventricular volumes and EF—may represent a valuable adjunctive component without additional imaging examinations. Consequently, future standardization of acquisition protocols, segmentation methods, and reproducibility criteria could further enhance the clinical utility of integrated morpho-functional CCT assessment within multimodality cardiovascular imaging pathways.

## 9. Conclusions

The determination of EF and ventricular volumes by CCT has reached a high level of technical maturity and clinical validation. These data suggest the complementarity of this imaging modality. CCT is not recommended to, and does not intend to, replace routine echocardiography or supplant CMR as the isolated gold standard for functional analysis. EF measurement by CCT represents an adjunctive tool: pathophysiological information of clinical importance obtainable at no extra cost and simultaneously when the investigation is prescribed for primary anatomical evaluations, such as the exclusion of CAD, the planning of transcatheter interventions or thoracic staging in the cardio-oncology setting. The progressive integration of AI will likely continue to automate and streamline this extraction process, and CCT has been shown to be a synergistic tool, enhancing personalized diagnostic pathways without replacing established functional imaging modalities.

## Figures and Tables

**Table 2:** Technological Evolution of Cardiac Computed Tomography: Comparison Between Historical and Modern Wide-Detector Scanners for Volumetric and Ejection Fraction Assessment.

Parameter	**Historical CT Scanners (e.g., 64-slice)**	**Modern Wide-Detector CT Scanners (e.g., 256/320-slice, 16 cm)**
Acquisition strategy & *Z*-axis coverage	Requires multiple rotations of the X-ray tube and data acquisition over several cardiac cycles to cover the entire longitudinal extension of the heart (approximately 12–14 cm) [1,24,38,72].	*Z*-axis coverage of up to 16 cm allows for whole-heart volumetric imaging in a single gantry rotation and within a single heartbeat [24,28,30,38].
ECG synchronization protocol	Retrospective protocols are necessary to reconstruct the entire R-R cycle in 5–10% intervals [3,23,25,30,46,74]	Allows for prospective acquisitions limited only to the systolic and diastolic phases [12,13].
Radiation exposure	Exposes the patient to considerable radiation doses [24,31,46,61].	Effective dose can be reduced below 1–2 mSv [24,31,33,61].
Susceptibility to artifacts	Prone to stair-step artifacts in cases of heart rate variability or arrhythmias [38,46].	Absence of spatial misregistration and mitigation of stair-step artifacts. However, extreme variations in the R-R cycle length can still require complex motion-correction algorithms [33,38]
Impact on volumetric and EF Accuracy	Artifacts can irreparably invalidate the calculation of EDV and ESV [29,38,46].	Guarantees volumetric measurements of the left ventricular chamber of unprecedented precision, returning highly accurate SV and EF values [23,24,25,33,46].

**Table 3:** Head-to-Head Comparison of Imaging Modalities for Volumetric and Ejection Fraction Assessment.

**Parameter**	**Two-Dimensional Transthoracic Echocardiography**	**Cardiac Magnetic Resonance**	**Cardiac Computed Tomography**
Clinical Role	Most accessible and widely adopted first-line modality [9,19,46].	Absolute reference gold standard for functional analysis [2,16,19,49,79].	Diagnostic tool when functional analysis is integrated with primary anatomical indications [12,19,22,33].
Temporal Resolution	Extremely high: 10–11 ms (equivalent to 90–100 fps) [8,17,19].	High: 20–40 ms (acquiring between 30 and 50 phases per cardiac cycle) [3,31,36,69].	Lower: 66–140 ms, physically constrained by the gantry rotation speed [17,24,33].
Volumetric Assessment (EDV/ESV)	Systematic underestimation of true volumes due to geometric assumptions and apical foreshortening artifacts [14,19,46,79].	High precision due to intrinsic contrast between hyperintense blood pool and medium-signal intensity myocardium [2,19,49].	Overcomes geometric assumptions, generating consistently higher EDV and ESV values than 2D-TTE. May slightly overestimate ESV and potentially underestimate EDV compared to CMR [19,23,61,82].
Ejection Fraction	Based on the biplane method of disks (modified Simpson’s rule) [1,19].	Reference method characterized by high reproducibility [2,15,19].	Slight systematic underestimation (on average by 2–4%) compared to CMR [19,65,79,82].
Primary Limitations	Operator dependence, assumption of standard geometric shape, and suboptimal acoustic windows (e.g., obese patients, severe emphysema, or mechanical ventilation) [1,14,19,46].	Precluded in patients with non-MRI-conditional implantable electronic devices, severe claustrophobia, or marked dyspnoea preventing prolonged supine positioning [16,19,22].	Exposure to ionizing radiation, toxicity of iodinated contrast media, and reliance on heart rhythm stability (severe arrhythmias invalidate ECG-gating) [19,21,22,46].

## Data Availability

No new data were created or analyzed in this study. Data sharing is not applicable to this article.

## Abbreviations

The following abbreviations are used in this manuscript:

AI	Artificial Intelligence
CAD	Coronary Artery Disease
CCT	Cardiac Computed Tomography
CRT	Cardiac Resynchronization Therapy
CTP	Computed Tomography Perfusion
CTRCD	Cancer Therapy-Related Cardiac Dysfunction
CT	Computed Tomography
CMR	Cardiac Magnetic Resonance
ECV	Extracellular Volume
EDV	End-Diastolic Volume
ESV	End-Systolic Volume
EF	Ejection Fraction
GLS	Global Longitudinal Strain
HF	Heart Failure
HFmrEF	Heart Failure with mildly reduced Ejection Fraction
HFpEF	Heart Failure with preserved Ejection Fraction
HFrEF	Heart Failure with reduced Ejection Fraction
ICD	Implantable Cardiac Device
LA	Left Atrial
LIE	Late Iodine Enhancement
LV	Left Ventricle
LVEF	Left Ventricle Ejection Fraction
MACE	Major Adverse Cardiovascular Events
RV	Right Ventricle
RVEF	Right Ventricle Ejection Fraction
SV	Stroke Volume
TAVI	Transcatheter Aortic Valve Implantation
TTE	Transthoracic Echocardiography
ACC	America College of Cardiology
AHA	American Heart Association
ASE	American Society of Echocardiography
CCTA	Coronary Computed Tomography Angiography
CI-AKI	Contrast-Induced Acute Kidney Injury
CNNs	Convolutional Neural Networks
ECG	Electrocardiogram
ESC	European Society of Cardiology
HER2	Human Epidermal Growth Factor Receptor 2
LOE	Level of Evidence
MDCT	Multidetector Computed Tomography
MUGA	Multiple Gated Acquisition (scan)/Radionuclide ventriculography
Nt-proBNP	N-terminal pro-b-type natriuretic peptide
PACS	Picture Archiving and Communication System
SPECT	Single-Photon Emission Computed Tomography
SSFP	Steady-State Free Precession
